# Experience-Regulated Neuronal Signaling in Maternal Behavior

**DOI:** 10.3389/fnmol.2022.844295

**Published:** 2022-03-24

**Authors:** Ileana Fuentes, Yoshikazu Morishita, Sofia Gonzalez-Salinas, Frances A. Champagne, Shusaku Uchida, Gleb P. Shumyatsky

**Affiliations:** ^1^Department of Genetics, Rutgers University, Piscataway, NJ, United States; ^2^Department of Psychology, University of Texas at Austin, Austin, TX, United States; ^3^SK Project, Medical Innovation Center, Kyoto University Graduate School of Medicine, Kyoto, Japan

**Keywords:** synapse, maternal care, gene tanscription, synaptic transport, depression, postpartum, postpartum depression, microtubules

## Abstract

Maternal behavior is shaped and challenged by the changing developmental needs of offspring and a broad range of environmental factors, with evidence indicating that the maternal brain exhibits a high degree of plasticity. This plasticity is displayed within cellular and molecular systems, including both intra- and intercellular signaling processes as well as transcriptional profiles. This experience-associated plasticity may have significant overlap with the mechanisms controlling memory processes, in particular those that are activity-dependent. While a significant body of work has identified various molecules and intracellular processes regulating maternal care, the role of activity- and experience-dependent processes remains unclear. We discuss recent progress in studying activity-dependent changes occurring at the synapse, in the nucleus, and during the transport between these two structures in relation to maternal behavior. Several pre- and postsynaptic molecules as well as transcription factors have been found to be critical in these processes. This role reflects the principal importance of the molecular and cellular mechanisms of memory formation to maternal and other behavioral adaptations.

## Overview

Parental care is an example of social affiliative behavior that is critical for the survival of offspring through its role in reproduction. Parental care exists in a wide range of animals, from invertebrates to fish and higher vertebrates, being abundantly present in birds and mammals. Parental care plays a fundamental role in keeping offspring safe and healthy (Numan and Insel, 2003; Numan et al., 2006). This role requires a high degree of physiological and behavioral plasticity in response to changing environmental cues and threats that are facilitated by changes in brain systems that regulate social behavior, stress responsivity, fear responses, and learning and memory. Lab-based studies of maternal behavior have explored these systems using rats and mice, with measures of maternal care including nest building, pup retrieval, pup licking, nursing, and defense of the young (Numan and Insel, 2003; Kuroda et al., 2011; Gammie, 2013). This literature has highlighted the behavioral transitions that females undergo following parturition and/or exposure to pups that results in a shift from pup aversion to pup-directed behavior (Cosnier, 1963; Rosenblatt, 1967; Fleming, 1989; Lonstein and De Vries, 2000; Numan et al., 2006; Kuroda et al., 2011; Stolzenberg and Mayer, 2019).

However, these behavioral transitions may come with a cost. Pregnancy and postpartum are associated with an increased risk of developing depressive symptoms (Woody et al., 2017; Qiu et al., 2020). Postpartum mental disorders in humans and maladaptive maternal behavior in animals are associated with significant adverse and long-term effects for both mother and offspring. Postpartum mental disorders, such as postpartum depression (PPD), are characterized by anxiety, depression, and poor maternal care. PPD is an episode of major depressive disorder (MDD) but has its own unique characteristics, including differences in the manifestation of depression with onset during pregnancy vs. postpartum onset (Gavin et al., 2005; Gaynes et al., 2005; Meltzer-Brody, 2011; Pawluski et al., 2017; Qiu et al., 2020). PPD affects 15%–25% of mothers worldwide. The impact of maintaining and improving a healthy mental state of the mother following the delivery is profound and far-reaching. The design of pharmacological and behavioral treatments of PPD would greatly benefit from a better understanding of the neurobiological mechanisms of maternal care (Numan and Insel, 2003; Qiu et al., 2020). This major gap in knowledge is partially due to the limited number of animal models of PPD, linked to either genetic or environmental causes (Pawluski et al., 2017; Qiu et al., 2020). While we know some of the molecular and cellular events as well as neural circuits involved in normal maternal care, the specific changes that underlie maternal dysfunction in animals and PPD in humans remain unclear.

**Figure 1:**
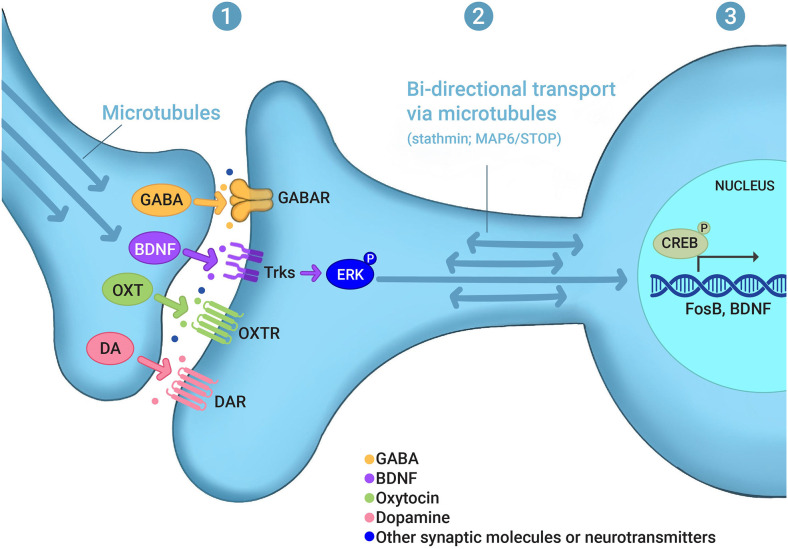
Experience-dependent signaling in maternal behaviors. Evidence discussed in this review indicates that experience- or activity-dependent signaling may occur **(1)** at synapses, affecting the localization of pre- and postsynaptic receptors and other synaptic proteins as well as changes in the post-translational modifications of synaptic proteins, such as GABA, BDNF, oxytocin (OXT), and dopamine (DA); **(2)** at the level of the bidirectional transport between synapses and the nucleus, which involves microtubule and microtubule-regulating proteins, such as stathmin and MAP6/STOP; and **(3)** at the level of gene transcription in the nucleus, which involves, for example, CREB as a transcription factor and *FosB* and *BDNF* as gene targets. (P) in ERK-P and CREB-P denotes protein phosphorylation. For other abbreviations see the main text.

Integrating the mechanisms that underlie the plasticity associated with learning and memory and the behavioral transitions associated with maternal behavior may generate unique insights into this critical reproductive behavior. In this review, we explore the hypothesis that experience-dependent signaling networks that control synaptic and nuclear function may be an important component of the molecular basis of maternal care. This activity-dependent signaling may occur: (1) at synapses, affecting the localization of pre- and postsynaptic receptors and other synaptic proteins as well as changes in the post-translational modifications of synaptic proteins; (2) at the level of the bidirectional transport between synapses and the nucleus; and (3) at the level of gene transcription in the nucleus (Figure 1). The molecular and cellular mechanisms underlying maternal care are very diverse, which is illustrated by the fact that maternal dysfunction in animals and PPD in humans are clearly multifactorial, with several genes associated with disrupted maternal care (Numan and Insel, 2003; Brummelte and Galea, 2010b; Di Florio and Meltzer-Brody, 2015; Gammie et al., 2016; Li and Chou, 2016; Stolzenberg and Champagne, 2016; Feldman et al., 2019; Froemke and Young, 2021). Importantly, a significant number of these genes are known to be part of activity-dependent signaling, synaptic function, and changes in transcription in various systems and behaviors, particularly in memory processes. With some exceptions (e.g., long-term potentiation and social learning), we propose that activity-dependent and memory-associated intracellular signaling pathways, but not memory processing *per se*, serve as critical mechanistic regulators of maternal care.

## Brain Anatomy of Maternal Behavior

Here we discuss the molecular mechanisms of maternal care in mice and rats, as the neurobiology of parenting behavior has been primarily studied in mothers of these two common model organisms (Kuroda et al., 2011; Dulac et al., 2014; Stolzenberg and Mayer, 2019). The molecular and cellular changes that are the focus of this review occur within the context of specific brain regions that have been implicated in maternal behavior. Experience-dependent changes have been described in specific brain areas during maternal care, including the medial preoptic area (mPOA) of the hypothalamus, ventral bed nucleus of stria terminalis (BNST), ventral tegmental area (VTA), nucleus accumbens (NAc), ventral pallidum, lateral septum and medial amygdala (Champagne et al., 2001, 2003, 2004; Francis et al., 2000, 2002; Numan and Insel, 2003; Tsuneoka et al., 2013; de Moura et al., 2015; Ray et al., 2015; Stamatakis et al., 2015; Gammie et al., 2016; Alsina-Llanes and Olazábal, 2020; Qiu et al., 2020). The mPOA-VTA-NAc network, which is part of the motivational circuit, is regulated by projections from the paraventricular nucleus of the hypothalamus (PVN), lateral habenula (lHb), and the dorsal raphe nucleus (Kohl et al., 2017). These brain regions are part of the excitatory system of maternal behavior (Numan and Insel, 2003). The medial amygdala has been implicated in maternal behavior through its inhibitory role in pup approach behavior (Cosnier, 1963; Rosenblatt, 1967; Numan and Insel, 2003; Lévy and Keller, 2009; Stolzenberg and Mayer, 2019). Olfactory cues from the pups are processed by the accessory and main olfactory bulbs which increase the activity of the medial amygdala in virgin females and promotes pup avoidance.

Cortico-limbic and in particular threat-related brain areas involved in anxiety and depression in humans and in affective disturbances in rodents also exhibit significant influence on maternal behavior; however, the neural mechanisms of this regulation remain obscure. In rodents, recent work suggests that the hippocampus (HPC), medial prefrontal cortex (mPFC), and basolateral amygdala (BLA) are involved in regulating maternal care (Fleming et al., 1980; Fleming and Korsmit, 1996; Walsh et al., 1996; Lee et al., 2000; Mattson and Morrell, 2005; Afonso et al., 2007; Lee and Brown, 2007; Pawluski and Galea, 2007; Maguire and Mody, 2008; Martel et al., 2008; Leuner and Gould, 2010; Numan et al., 2010; Pereira and Morrell, 2020). In humans, functional magnetic resonance imaging (fMRI) has shown that postpartum anxiety and depression in mothers are characterized by abnormal functional connectivity in both resting state and in response to infant cues in several brain regions, including the amygdala, anterior cingulate cortex, PFC, and HPC (Silverman et al., 2007; Leuner et al., 2010; Moses-Kolko et al., 2010; Meltzer-Brody, 2011; Schiller et al., 2015; Pawluski et al., 2017). Moreover, the HPC, amygdale, and PFC are some of the overlapping brain regions involved in memory, postpartum states, and depression in humans (Leuner and Shors, 2006, 2013). Importantly, even though only a few animal models of peripartum affective disorders exist, they show changes in synaptic structure, synaptic plasticity, synaptic proteins, and nuclear function (gene transcription) in same brain areas that have altered fMRI activity in mothers with PPD compared with healthy mothers (Pawluski et al., 2017). Maternal care impacts neuro genesis as well as dendritic spine morphology in the HPC and several other brain areas (Pawluski et al., 2016; Duarte-Guterman et al., 2019). Hippocampal long-term potentiation (LTP) is increased in mothers, an effect that is abolished by gestational stress (Pawluski et al., 2016). Moreover, GABA_A_ receptors, whose expression in the HPC is modulated during pregnancy, are involved in pup retrieval and affective behaviors in the postpartum but not in virgin females (Maguire and Mody, 2008), suggesting a specific role for the HPC and cortico-limbic threat circuits in maternal behavior.

## Cell-Type Specificity of Activity-Regulated Events in Maternal Behavior

While it may be challenging to distinguish between cells that are “merely” activated by general activity and cells inducing a behavior, significant progress has been made in establishing the causal role of specific cells and circuits in behavior. Systems neuroscience has evolved with powerful methods of studying brain circuits by fast and slow time scale manipulation (e.g., optogenetics, chemogenetics) and imaging *in vivo* (Aston-Jones and Deisseroth, 2013; Bruchas and Roth, 2016; Kim et al., 2017; Nectow and Nestler, 2020). It is worth noting that if a group of cells is activated by a behavior but the manipulations used on these cells do not produce an effect on the behavior, this is not necessarily evidence that the cell population is not important for this particular behavior. Rather, it is likely the case that our approaches to testing the functionality of these cells are not sensitive enough to fully understand the processes underlying their function. To address these issues, a more nuanced approach might be necessary, which in addition to probing the circuitry-level activity and electrophysiological properties of cells, also examines the mechanisms of transcription, translation, receptor function, trafficking, and other intracellular processes (Shen et al., 2022). Combining these approaches will allow modern neuroscience to distinguish between molecular cause and effect in brain function and various behaviors, including maternal care.

Immediate early gene (IEG) activation following a mother’s interaction with pups have contributed to mapping brain regions involved in maternal care in rodents (Lonstein et al., 2000; Numan, 2007; Tsuneoka et al., 2013), but the identity of the cell types has only recently been explored. In the mPOA more than 75% of the cells that become active during maternal care are GABAergic (Tsuneoka et al., 2013). These GABAergic cells express estrogen receptor alpha, galanin, neurotensin, and tachykinin2 (Lonstein et al., 2000; Tsuneoka et al., 2013). Chemo- and optogenetic approaches have recently shown that the activation of mPOA cells expressing the estrogen receptor alpha induces pup approach and retrieval (Fang et al., 2018; Wei et al., 2018). The activation of galanin-expressing cells that project from the mPOA to the peri aqueductal gray matter or VTA promotes grooming and motivation to seek pups (Kohl et al., 2018). Interestingly, ablation of the galanin-expressing cells in the mPOA leads to the reduction of several forms of maternal care but activation of these cells improves pup grooming only, suggesting a complex role of these cells in maternal care (Wu et al., 2014). GABAergic mPOA cells, different from those expressing the estrogen receptor alpha, might be involved in nest building behavior but their identity is unclear (Li et al., 2019). Outside of the mPOA, inhibitory neurons in the arcuate nucleus of the hypothalamus that express the agouti-related neuropeptide (AGRP) form synapses on mPOA cells and their activation decreases nest building (Li et al., 2019). In the medial amygdala, the activation of GABAergic cells, but not of glutamatergic ones, promotes pup grooming and to a lesser extent pup retrieval (Chen et al., 2019).

Similar to chemo- and optogenetic studies, fiber photometry illustrates that pup sniffing or grooming increase intracellular calcium in galanin-expressing mPOA cells while pup approach and retrieval increase calcium signal in mPOA cells expressing the estrogen alpha receptor (Fang et al., 2018; Kohl et al., 2018; Wei et al., 2018). Intracellular calcium in GABAergic cells in the medial amygdala is also increased during pup grooming (Chen et al., 2019).

## Maternal Behavior and Learned Responses

The maternal brain, as we have learned from human and rodent studies, is highly dynamic and susceptible to both internal and external influences (Olazabal et al., 2013a; Stolzenberg and Champagne, 2016; Stolzenberg and Mayer, 2019). The expression of maternal behavior relies on the hormonal state of the mother and sensory cues coming from the offspring. The maternal brain is continuously processing external sensory information and must adjust its activity to meet the demands of the offspring (Olazabal et al., 2013a, b). This plasticity is displayed at both intra- and intercellular levels. Activity-dependent genes and proteins may play an important role at the early stages following initiation of neuronal activity elicited by the mother-offspring interaction and later provide the intracellular mechanism for encoding these experiences into long-term memory. Therefore, it may be instructive to compare activity-regulated processes emerging in maternal care to those that are well-established in memory. Memory processing is dynamic and plastic. In a somewhat similar manner, motherhood enhances the plasticity of the female brain, affecting neurogenesis, dendritic spine morphology, synaptic proteins, gene transcription, LTP, and memory (Pawluski and Galea, 2006; Leuner and Sabihi, 2016), and these processes are affected during perinatal stress and affective disturbance in animals and in peripartum depression in humans (Qiu et al., 2020). Activity-dependent synaptic and nuclear events have been described quite extensively for learning and memory, with several in-depth reviews on this topic (Klann and Dever, 2004; Alberini, 2009; Mayford et al., 2012; Nonaka et al., 2014; Ch’ng et al., 2015; Yap and Greenberg, 2018).

To begin an exploration of possible plasticity events occurring during maternal care, it is important to examine the role of synaptic plasticity including LTP. LTP is a widely accepted model of the cellular mechanisms of activity-dependent synaptic plasticity leading to memory formation (Bliss and Lomo, 1973; Siegelbaum and Kandel, 1991; Malenka and Nicoll, 1997; Stevens, 1998; Martin et al., 2000; Poo et al., 2016). Activity-dependent genes are critical for both LTP and memory processing and somewhat similar links can be expected between genes, synaptic plasticity, and maternal care. With daily pup exposure, virgin females learn to engage in the full repertoire of maternal care (Fleming and Rosenblatt, 1974). The sensory cues elicited by pups change the neural activity in the female brain. In particular, learned responses to pup’s auditory cues are critical for efficient maternal care. There is evidence for the role of maternal physiological state (virgin females vs. mothers) in creating a memory for ultrasonic vocalizations from pups (USVs; Lin et al., 2013). Previous experience with progeny may transform and shape initial stereotypical maternal care responses into a learned behavior, making these responses more adaptive to the current situation surrounding the mother and her progeny. In the postpartum period, the representation of pup calls in the primary somatosensory cortex increases and is later refined, likely due to activity-dependent plasticity elicited during nursing. Pup calls produce a stronger activation of the auditory cortex in mothers compared to virgin females, and the balance between excitation and inhibition is changed (Valtcheva and Froemke, 2019). The temporal association cortex receives inputs from the auditory cortex and exhibits activity-dependent changes that improve the discrimination of pup calls in mothers compared with females (Tasaka et al., 2020). Auditory-driven plasticity has also been found in the temporal association cortex (TeA) in mothers in response to USVs from pups. Tasaka et al. (2020) suggest that TeA processes USVs to support the memory of pup cries by the parents, somewhat similar to how TeA processes information for auditory memory in fear conditioning (Romanski and LeDoux, 1992; Quirk et al., 1997). Other work also indicates that maternal experience-dependent cortical plasticity is involved in the ability to retrieve pups (Lau et al., 2020). Plasticity is also related to a gene knockout of the *Mecp2*, which loss-of-function mutations cause the neuro developmental disorder Rett syndrome that has autistic features (Amir et al., 1999). Following up on this initial evidence that synaptic plasticity may be involved in maternal care, it would be important to study various forms of synaptic plasticity in relation to maternal experience.

Changes in dendritic spine morphology are another important activity-dependent cellular event. Changes in dendritic spines are believed to be a critical part of memory processing (Rogerson et al., 2014; Bailey et al., 2015; Dent, 2017). These changes are also consistently found during normal motherhood as well as in animal models of maternal stress and affective behaviors in the postpartum (Workman et al., 2013; Glasper et al., 2015; Pawluski et al., 2016; Duarte-Guterman et al., 2019; Sheppard et al., 2019). In addition, neurogenesis is affected, as cell proliferation and survival are decreased during gestation and early postpartum, and exogenous treatment with corticosterone reduces proliferation even further.

In addition to mother’s own experience in nursing, learned adaptations that improve maternal care include learning by “social transmission” from other experienced mothers (Schiavo et al., 2020; Carcea et al., 2021). Learning maternal care from more experienced females would improve survival of the progeny, and both wild and laboratory mice as well as rats prefer to rear their young in communal nests and nurse their own and other mothers’ pups (Branchi, 2009; Heiderstadt and Blizard, 2011; Weidt et al., 2014). This process is perhaps somewhat similar to the role of memory processes in generating social learning (Basu and Siegelbaum, 2015; Leblanc and Ramirez, 2020).

In addition to the similarities between the molecules regulating memory and maternal behavior, there is evidence that spatial memory is affected peripartum (Perani and Slattery, 2014). Pregnant rats perform better than virgin females in spatial memory tasks during the first two trimesters of pregnancy (Galea et al., 2000). However, maternal memory is impaired in the last trimester of pregnancy and after the delivery (Galea et al., 2000; Darnaudery et al., 2007), somewhat similar to studies in humans, showing that some memories are diminished during pregnancy and after parturition (Glynn, 2010). Therefore, it is possible that an increase in memory processes devoted to maternal care takes a toll on other brain functions, including spatial memory, as pregnancy, giving birth and caring for the progeny require and consume significant energetic resources.

## Why Are Activity-Regulated Events at The Molecule Level Important for Maternal Behavior?

Learning and memory depend on neuronal plasticity originating at the synapse following an exogenous stimulus and requiring gene transcription in the nucleus to persist. RNA and protein products following these transcription events are transported back to synapses strengthening synaptic connections. While we are beginning to understand activity-regulated processes and how synapse-nucleus communication supports long-term neuronal plasticity as well as learning and memory, the role of these processes in maternal behavior remains unclear. Synaptic and nuclear changes, as well as microtubule-mediated transport connecting them, are known to be activity-regulated, these processes however are just some of many activity-regulated intracellular events. Other processes include changes in the blood flow, spine dynamics, synaptic connections, intracellular movement of organelles *via* synaptic and nuclear trafficking, and changes in protein structure and function. This is clearly not a full list as we are learning that some of the molecular and cellular events that were once considered stable are in fact dynamic in the adult mature brain [for example, neurogenesis and microtubule stability (see Section “Microtubule-Mediated Transport in Maternal Care”)]. It is important to note that it is challenging to completely separate the basal processes at synapses and in the nucleus from activity-dependent events, as most of the genes and proteins involved are active not only as a result of activity but to a certain degree also in the basal “naïve” state. Alternative explanations for the molecular basis of maternal care are also important to consider, such as dysregulation of synaptic function in general, impairment of neurotransmission (which may influence activity-dependent signaling) and changes in early development.

In the following sections, we will review some examples of activity- and experience-dependent changes at synapses, in the nucleus and in microtubules, which mediate the synapse-nucleus communication.

## Synaptic Molecules in Maternal Care

Complex behaviors and memory are guided by the interaction of molecules in the pre- and post-synaptic sites of neurons (Bailey et al., 2015; Chanaday and Kavalali, 2018; Monday et al., 2018). Biochemical changes at the level of neurotransmitters and neuromodulators would be expected to occur during pregnancy, the postpartum period and interactions with the progeny. Indeed, pup approach, sniffing, retrieval and grooming increase intracellular calcium in the mPOA and medial amygdala (Fang et al., 2018; Kohl et al., 2018; Wei et al., 2018; Chen et al., 2019). Together with work showing that ERK phosphorylation (ERK kinase is involved in the transmission of signals from synapses to the nucleus) is increased following the interaction with pups (Kuroda et al., 2007), these studies suggest that maternal care initiates calcium-dependent signaling cascades, which often lead to activity-dependent intracellular changes as is well documented for memory processes. Overall, the mechanism by which maternal motivation happens may be explained by the interaction between hormones and neurotransmitters, with both regulated in experience-dependent manner.

### Neurotransmitters Involved in Maternal Behavior and Postpartum Period

The flexibility of neuronal networks includes both the plasticity of excitatory and inhibitory synapses (Barberis, 2020). Changes in neurotransmitter release happen during pregnancy and the postpartum period. Glutamate is the most common excitatory neurotransmitter, it binds to the receptors for NMDA (N-methyl-d-aspartate) and AMPA (α-amino-3-hydroxy-5-methyl-4-isoxazole propionic acid). Activated NMDA receptors flux calcium, thereby inducing multiple calcium-dependent signaling pathways, including calcium-dependent kinases (CaMKII and CaMKIV), protein kinase A (PKA), mitogen-activated protein kinases (MAPKs) and calcium-dependent phosphatase calcineurin, which phosphorylate or dephosphorylate their substrates at the synapse or in the nucleus. Glutamate and γ-aminobutyric acid (GABA) are critical for memory processing (Luscher and Malenka, 2012). They were also shown to be involved in maternal care (Zhao and Gammie, 2014). Glutamate and GABA are upregulated in the mouse lateral septum postpartum (Zhao and Gammie, 2014). mRNA of the GluA3 and GluN2B of AMPA and NMDA receptors is increased in rat mothers and injections in the mPOA of CNQX, an AMPA receptor antagonist, and MK-801, an NMDA receptor antagonist, reduce maternal behavior in rat mothers (Okino et al., 2020).

#### GABA Receptor and Postpartum-Specific Maternal Behavior Deficits

Plasticity in mothers starts in pregnancy, and the δ subunit of the GABA_A_ receptor subtype (GABARδ) is involved in experience-dependent changes peripartum (Maguire and Mody, 2008). Mothers with a knockout of the GABA_A_Rδ (*Gabrd^−/−^*), which leads to a deficiency in synaptic function in the dentate gyrus of the HPC, neglect pups and have a decrease in pup survival. There are naturally occurring changes in GABAR plasticity during pregnancy; whole-cell patch recordings performed on the dentate gyrus granule cells show that tonic inhibition is decreased in wildtype pregnant mice compared to virgins and mothers. However, tonic inhibition in mice lacking the GABA_A_R δ subunit shows no changes across virgin, pregnant, and postpartum females. These data highlight the importance of changes in neuronal activity across different stages of the peripartum period since *Gabrd^−/−^*mice show behavioral deficits at the postpartum. *Gabrd^−/−^*mice exhibit depressive-like behavior and inability to take care of their progeny while no changes are observed in virgin *Gabrd^−/−^* mice. The neurosteroid allopregnanolone, a positive allosteric modulator of GABA’s action at GABA_A_R (Pinna, 2020), has helped to determine the role of GABARδ subunit through the peripartum period. Because of the presence of the GABA_A_R δ which allows neurosteroid sensitivity, allopregnanolone can enhance the tonic GABAergic inhibition mediated by GABA_A_Rs. Deletion of GABARδ leads to altered neuronal excitability due to lack of sensitivity to allopregnanolone (Maguire et al., 2009). This suggests a mechanism for how GABARδ exerts a homeostatic effect on neuronal activity during the peripartum period (Maguire et al., 2020).

Similar to the *Gabrd^−/−^* mice described above, deficient maternal care and affective (anxiety- and depressive-like) behaviors are observed in postpartum but not virgin female mice that lack the K ^+^/Cl^−^ co-transporter 2 (KCC2) specifically in neurons expressing the corticosterone-releasing hormone (CRH; Melon et al., 2018). Among several roles of KCC2, its increased expression in mature neurons lowers [Cl^−^]_i_, leading to an influx of Cl^−^ ions and hyperpolarizing responses upon GABA_A_R activation. KCC2 is important for the GABAergic regulation of CRH neurons in the paraventricular nucleus of the hypothalamus (PVN), as mice lacking KCC2 specifically in the CRH-positive neurons exhibited abnormal postpartum affective and maternal behaviors.

#### Dopamine (DA), Reward, and Maternal Behavior

DA has been intensively studied in maternal care and found to be important for the function of several major maternal brain areas, including the mPOA, ventral pallidum, and NAc (Numan et al., 2005a). As part of the reward system, DA is involved in how mothers respond to their offspring and find their young rewarding (Numan and Stolzenberg, 2009; Pereira and Morrell, 2010; Rincon-Cortes and Grace, 2020). DA is synthesized in both the VTA and substantia nigra and then transported by neuronal projections to the NAc, mPFC, and amygdala. Extracellular DA is increased in the mother rat NAc associated with pup licking and grooming (Champagne et al., 2004). DA transporter binding and DA receptors D1 and D3 are also increased. Transcription of the tyrosine hydroxylase (TH) and possibly other DA-related genes, is activity-dependent and is regulated by changes in membrane potential and intracellular Ca^2+^ (Aumann and Horne, 2012). Activity- and Ca^2+^-dependent regulation of TH expression is critical, as this protein activity is the rate-limiting enzyme in DA synthesis and is, therefore, a key orchestrator of cellular DA levels. An interesting possibility exists that dopamine may regulate AMPAR trafficking, as activation of dopamine receptors leads to synaptic insertion of the AMPAR (Wolf, 2010). Although studies on the hormonal and non-hormonal basis of maternal behavior have focused primarily on hypothalamic regions such as the mPOA, extensive neural circuitry contributes to all complex behavioral phenotypes. It has been suggested that the reward system is involved in the process by which the mother establishes and maintains a relationship with pups (Hauser and Gandelman, 1985). Indeed, it was shown that some of the neurons activated during nurturing behavior in the mPOA project to the VTA, which plays a central role in the reward system in the brain (Numan and Numan, 1997). Furthermore, dopaminergic neurons that project from the VTA to the NAc are known to be involved in the control of the expression of maternal behaviors (Keer and Stern, 1999; Numan et al., 2005b; Numan and Stolzenberg, 2009) and contribute to the elicitation of pleasure associated with the expression of behaviors, thereby supporting the enhancement and maintenance of further behaviors. It has been reported that the number of c-Fos positive cells in the VTA increases during maternal behaviors (Numan and Numan, 1997; Matsushita et al., 2015), and pathway-specific manipulations have also revealed that dopaminergic cells in the VTA are activated during approach and retrieval of pups (Fang et al., 2018). As mentioned above, dopamine release in the ventral striatum and NAc is elevated in nursing females interacting with pups (Hansen et al., 1993; Champagne et al., 2004), and dopaminergic projections from the VTA to NAc are strengthened in females providing a high level of maternal care (Shahrokh et al., 2010). These activity-dependent changes following neuroplasticity may support the long-term strengthening of maternal behavior.

Other neuromodulators known to be released in activity-dependent manner, such as norepinephrine, which is a ligand of the adrenergic receptor, are also involved in maternal behavior. Disruption of the *dopamine beta-hydroxylase* (*Dbh*) gene that does not allow synthesis of the norepinephrine and epinephrine leads to lower pup survival rate and poor ability of *Dbh^−/−^* mothers and virgin females to retrieve pups to the nest (Thomas and Palmiter, 1997). In rats manipulating norepinephrine release in vBNST and mPOA leads to deficits in pup retrieval (Smith et al., 2012).

### Hormones and Peptides

Sustained maternal care is critical for the survival of the progeny and hormonal changes help to trigger maternal motivation during pregnancy and postpartum. In mammals, maternal behavior is highly regulated by hormonal changes that fluctuate significantly during the peripartum period (Pawluski et al., 2017). In general estradiol, progesterone, and corticosterone increase during pregnancy and decrease around the time of labor or parturition (Duarte-Guterman et al., 2019). In humans, pregnancy and giving birth bring about not only physiological but also psychological changes, which are dependent on hormonal regulation of the peripartum (Olza et al., 2020; Thul et al., 2020). Studies in rodents show that estrogen and progesterone play a critical role in triggering maternal responses and interfering with their function impairs pup retrieval, licking, and nursing (Stolzenberg and Champagne, 2016).

Prolactin is also involved in maternal care, and progesterone and prolactin may interact in the mPOA to mediate maternal behavior. To study the role of hormones in motherhood, a treatment with pregnancy-like regimen of progesterone can be useful to mimic the natural process of hormone release during peripartum period. Treating female rats with this progesterone regimen for 10 days causes a reduction in the expression of the prolactin receptor mRNA in the mPOA (Bridges and Hays, 2005). Deletion of prolactin receptors from GABA neurons leads to impairment in maternal motivation; prolactin receptor gene knockout mothers become slower in pup retrieval, suggesting a link between hormones and GABAergic neurons in maternal behaviors (Swart et al., 2021).

The peptide oxytocin is another critical molecule for maternal care. It is synthesized in the hypothalamic neurons, packaged into dense-core vesicles, and transported into dendrites and axons for release (Froemke and Young, 2021). Oxytocin increases during pregnancy with a peak during and immediately following birth to support uterine contractions and initiate milk ejection during nursing (Rilling and Young, 2014; Thul et al., 2020). Oxytocin also plays a major role in the psychological experiences in the maternal state of women. Oxytocin together with dopamine activates specific neural pathways, to stimulate nurturing and bonding with the progeny. A systematic review of the human literature on mothers with PPD shows that most of the studies suggest an inverse relationship between plasma oxytocin levels and depressive symptoms (Thul et al., 2020). Oxytocin is released in an activity-dependent manner both in the brain and blood stream: birth and suckling in the lactating animal trigger oxytocin release inside various brain regions, which has been extensively studied (Jurek and Neumann, 2018; Grinevich and Neumann, 2021). In addition to oxytocin, oxytocin receptor, upon binding to oxytocin (as a result of an activity or experience), can activate multiple intracellular signaling cascades to promote *de novo* RNA and protein syntheses (Grinevich and Neumann, 2021). A recent study showed the role of the oxytocin expressed in cells expressing another hypothalamic neuropeptide, melanin concentrating hormone (MCH), in the control of maternal care and affective behaviors (Phan et al., 2020). The oxytocin receptor gene knockout limited to the MCH-expressing neurons increases depressive-like behavior in sexually naïve females and decreases depressive-like behavior in mothers. These behavioral changes seem to be associated with synaptic plasticity in the reward and fear circuits based on Arc expression, providing another example of experience-dependent changes in maternal care.

CRH and corticosterone are also important to consider when describing experience-dependent changes in maternal behavior. In addition to the KCC2 gene knocked out specifically in the CRH-positive cells described earlier in this review (Melon et al., 2018), work in prairie vole mothers suggests the role for the CRH system in the experience-dependent development of affective postpartum behaviors (Bosch et al., 2018). Abnormal changes in the level of corticosterone during pregnancy and the postpartum may contribute to PPD, and chronic corticosterone treatment has been used to induce postpartum malfunction in rats and mice, leading to deficits in neurogenesis and spine formation, dynamic cellular events that are experience-dependent (Brummelte et al., 2006, 2012; Brummelte and Galea, 2010a; Maguire and Mody, 2016).

### Brain-Derived Neurotrophic Factor

Neurotrophins are well established stimulators of neuronal IEG response and they play a major role in memory and depression (Yang et al., 2020). Synthesis and release of the brain-derived neurotrophic factor (BDNF) is regulated by neuronal activity (Lu, 2003; Cunha et al., 2010), therefore activity-dependent events may involve this neurotrophin during mother-progeny interactions. Indeed, maternal care strongly modulates BDNF expression in rodents (Liu et al., 2000; Branchi et al., 2013). The disruption of the BDNF signaling in the oxytocin neurons leads to reduced maternal care in mice (Maynard et al., 2018). *Bdnf* deletion decreases maternal behaviors in virgin females and mothers; knock-out females show harmful behaviors towards the pups including biting (Maynard et al., 2018). The authors suggest that BDNF could be a modulator of sex-specific social behaviors and be a new activity-dependent molecule critical for oxytocin neuron function (Maynard et al., 2018). Chronic unpredictable stress (CUS) applied after giving birth produces affective (depressive-like) behaviors, which are accompanied by a decrease in the *Bdnf* mRNA and protein levels in the mPFC of mothers (Liu et al., 2020). The *Bdnf* gene knockout in the mPFC also leads to changes in FoxO1 expression, which was previously implicated in major depressive disorders (Liu et al., 2020). As CUS and other stress procedures are known to alter maternal behavior (Leuner et al., 2014; Maguire and Mody, 2016), it is probable that the reduction in *Bdnf* and FoxO1 might also be involved in these behavioral disturbances. Moreover, female rats exposed to opium during pregnancy showed reduced maternal behaviors in the postpartum period that were accompanied by decreased BDNF expression in the HPC (Rezaei et al., 2021). Supporting the hypothesis that activity-dependent events produced by bouts of maternal care involve BDNF, mothers that spent more time grooming and licking pups have higher levels of BDNF in the HPC and NAc (Zhang et al., 2020). Oxytocin-induced regulation of BDNF may mediate the observed behavioral differences. BDNF binds the TrkB receptor to mediate many aspects of neural plasticity and behaviors (Lu, 2003). Disruption of the BDNF-TrKB signaling in oxytocin neurons leads to reduced maternal care in female mice, suggesting that BDNF could be a modulator of sex-specific social behaviors and be a new activity-dependent molecule critical for oxytocin function (Maynard et al., 2018). Human studies showing a relationship of genetic variations and methylation of *Bdnf* with parental rearing behaviors (Suzuki et al., 2011; Unternaehrer et al., 2015; Avinun and Knafo-Noam, 2017) highlight the necessity of more basic studies addressing the activity-dependent role of *Bdnf* during maternal care.

### Other Synaptic Proteins

Ephrin-A5 is another synaptosomal protein deletion of which leads to a decrease in maternal behavior and pup retrieval (Sheleg et al., 2017). In primary neurons Ephrin-A5 suppresses BDNF-induced ERK activity and BDNF-evoked neuronal IEG response, suggesting a role of Eph receptors in modulating gene expression. Opposite to IEGs, long-term ephrin-A5 application induces cytoskeletal gene expression of tropomyosin and actinin (Meier et al., 2011). These data suggest a possibility that Ephrin-A5 can regulate activity-regulated cellular and transcriptional changes in maternal behavior. Interestingly, another member of the Ephrin family, Ephrin-A2, is on the axon guidance ontology list in a DNA methylation study of PPD patients (Nakamura et al., 2019).

Experience-dependent maternal behavior can be regulated by proteins that act at the presynaptic active zone of the neuron. The presynaptic active zone protein CAST, which is a presynaptic release machinery-protein that acts as an anchor for neurotransmitters and neuromodulators was examined for its role in maternal behavior, using CAST knockout primiparous and multiparous females (Hagiwara et al., 2020). Primiparous CAST knockout females showed a decrease in crouching and nest building which were significantly improved with their second litter. CAST knockout virgin females failed to learn maternal behavior even after repeated exposure to newborn pups. The authors also found changes inaffective behaviors (sucrose preference) in pregnant but not virgin females. The CAST protein is distributed in the posterior pituitary, suggesting that it might regulate the release of oxytocin through the hypothalamus magnocellular neurons. However, there were no differences in oxytocin serum levels, suggesting that a different mechanism, other than hormonal pathway, is affecting CAST-dependent maternal behavior.

Neurotransmitter action is regulated by various molecules including the heterotrimeric G proteins of the Gq/11 family. Mice that lack the α-subunit in the forebrain show reduced nest building, pup retrieval, crouching and nursing (Wettschureck et al., 2004). The deficiency in maternal behavior is not due to a decrease in oxytocin release or prolactin production, since pituitary function is normal in Gαq/11 deficient mice.

However, other transmembrane proteins, such as glycoproteins, can act *via* oxytocin release and affect the maternal experience. For instance, the CD38 transmembrane glycoprotein with ADP-ribosyl cyclase activity is involved in maternal behavior through regulation of oxytocin secretion (Jin et al., 2007; Young, 2007). CD38 knockout multiparous female mice retrieve pups faster than CD38 knockout primiparous females. Both wildtype and CD38 knockout experienced mothers have an increase in oxytocin release from hypothalamus, however the basal level of plasma oxytocin of wildtype experienced dams is only slightly higher. Thus, CD38 regulation of oxytocin plays an important role in plasticity in experienced mothers.

A recent report has identified a new player in maternal care, the T-cell death-associated gene 51 (TDAG51). TDAG51 is a member of the pleckstrin homology-like domain family and was first identified as a pro-apoptotic gene in T-cell receptor-mediated cell death (Park et al., 1996). Members of this family of proteins are involved in the regulation of p53 and AKT signaling pathways. TDAG51 expression in pregnant mice was higher during the prenatal, parturition and postnatal periods compared to that in virgin female mice (Yun et al., 2019). TDAG51 knockout dams showed reduced pup retrieval and impaired nest building behavior, thus suggesting that the experience-dependent TDAG51 expression could be involved in maternal behavior.

## Microtubule-Mediated Transport in Maternal Care

Trafficking in both directions between synapses and the nucleus is critical for activity-dependent intracellular neuronal signaling. While the critical role of trafficking has been shown for many behavioral processes including learning and memory, how important it is for maternal care remains unclear. The first step in this process is the transmission of extracellular signals received by synapses to the nucleus to induce the corresponding changes in gene transcription (Cohen et al., 2015; Herbst and Martin, 2017; Uchida and Shumyatsky, 2018b; Parra-Damas and Saura, 2019). Trafficking in the opposite direction from the nucleus and cell body supplies synapses with various cargos, including synaptic vesicle precursors, neurotransmitter and neurotrophic factor receptors, other synaptic proteins, mRNAs, and organelles (Hirokawa et al., 2010).

Microtubules are one of the major cytoskeletal structures in neurons (Conde and Caceres, 2009), and microtubule-mediated trafficking is at the core of the signaling between synapses and the nucleus (Hirokawa et al., 2010). Recent work has shown that microtubules in the mature brain are dynamic, changing their stability in response to external events including those that lead to memory processing (Shumyatsky et al., 2005; Conde and Caceres, 2009; Jaworski et al., 2009; Fanara et al., 2010; Uchida et al., 2014; Uchida and Shumyatsky, 2015; Martel et al., 2016; Dent, 2017; Yousefzadeh et al., 2020). Similarly, the microtubule-associated machinery may be critical for experience-dependent intracellular changes during maternal care. Several studies have reported that proteins in the microtubule network are changed during pregnancy and the postpartum.

Expression of microtubule-associated protein 2 (MAP2), tau and glial fibrillary acidic protein (GFAP), and tau phosphorylation change in the rat hypothalamus, mPOA, HPC, frontal cortex, and cerebellum during pregnancy and the postpartum (Gonzalez-Arenas et al., 2012, 2014). Following pup exposure in both primiparous and multiparous female rats, the GFAP, a major molecule of the cytoskeleton network in astrocytes, is increased in mPOA astrocytes, whereas there is a decrease of GFAP in the medial amygdala and habenula (Featherstone et al., 2000).

The stathmin family of proteins are negative regulators of microtubule formation (Chauvin and Sobel, 2015). Stathmin in its unphosphorylated form binds tubulin dimers and, once phosphorylated, releases tubulin allowing microtubules to be formed. Stathmin has been shown to regulate innate and learned threat responses (Shumyatsky et al., 2005; Martel et al., 2012). Stathmin may serve as a molecular link between threat assessment and maternal behavior, as *stathmin^−/−^* mothers and virgin females are deficient in nest building, pup retrieval, and choosing a safe location for the nest in an open field (Martel et al., 2008). Stathmin phosphorylation changes in response to learning, in turn, causing microtubules to change their stability and synaptic microtubule-mediated transport (Uchida et al., 2014; Martel et al., 2016). It is possible therefore that stathmin may change its binding to tubulin, microtubule-destabilizing activity, and regulate microtubules in response not only to learning but also maternal events: pregnancy, the postpartum as well as maternal care, such as nest building, pup retrieval, and grooming.

Microtubule stabilizer MAP6/Stable Tubule Only Polypeptide (STOP) is another microtubule-associated protein. It binds to and stabilizes microtubules and induces nocodazole resistance and tubulin detyrosination (Bosc et al., 2003). Similar to MAP2, STOP/MAP6 stabilizes microtubules by bridging the binding between adjacent microtubules, which is regulated by calmodulin binding (Lefevre et al., 2013). STOP/MAP6 regulates synaptic function and maternal behavior providing another link between microtubules and maternal care (Andrieux et al., 2006). Whether STOP/MAP6 can change its activity during pregnancy and the postpartum is unknown but because it is regulated by calmodulin, it may be involved in experience-dependent intracellular events during maternal behavior.

In addition, a proteomics study on the mPFC in postpartum rats found that microtubule-related proteins represented 10% and those involved in microtubule-mediated synaptic transport and plasticity represent 19% of all proteins identified in the study (Volgyi et al., 2017).

Because of the critical role microtubules have in axon guidance (Gu et al., 2020), it is interesting to note that genes involved in axon guidance were among four ontology terms related to PPD that were found in a case control study of a DNA methylation analysis of PPD (Nakamura et al., 2019).

## Nuclear Events in Maternal Care

The nuclear events may alter social behaviors, such as maternal experience. Transcription factors can play a role in maternal behavior as they do in learning and memory as well as being disturbed in neurodegenerative and mental disorders (Deisseroth and Tsien, 2002; Greer and Greenberg, 2008; Ebert and Greenberg, 2013; Poo et al., 2016; Uchida and Shumyatsky, 2018a, b; Yap and Greenberg, 2018; Parra-Damas and Saura, 2019).

Extracellular regulated kinase (ERK)-mediated signaling is one of the best examined synapse-to-nucleus signal transduction networks in the central nervous system. In neurons, synaptic inputs activate the ERK cascade and in turn stimulated ERK can phosphorylate a variety of proteins which are involved in synaptic plasticity [i.e., LTP and long-term depression (LTD)], synaptogenesis as well as transcriptional and translational regulation (Kelleher et al., 2004; Thomas and Huganir, 2004), including IEG (see, for example, the ERK-FosB link below) as well as learning and memory (Impey et al., 1999; Sweatt, 2001; Satoh et al., 2011b). ERK was also found to play a critical role in regulating maternal behavior, with brain-specific ERK knockout mice (*Erk2* CKO mice) exhibiting deficiency in time spent crouching over the pups (Satoh et al., 2011a).

### Transcriptional Regulation

#### CREB

Since the discovery of the role of FosB in maternal care, several lines of evidence have shown an important role of transcription factors in maternal behavior. The cyclic adenosine monophosphate (cAMP) responsive element-binding protein (CREB) is one of the most studied transcription factors in memory and there is growing evidence for its role in maternal care. CREB regulates the expression of many genes and is involved in activity-regulated gene transcription (Deisseroth and Tsien, 2002; Cohen et al., 2015; Yap and Greenberg, 2018). CREB phosphorylation (pCREB) is critical for its transcriptional activity (West et al., 2002). The levels of pCREB in the mPOA are directly correlated to the strength of licking/grooming in lactating rats (Parent et al., 2017). Moreover, the number of cells immunostaining for pCREB (on Ser^133^) in the mPOA significantly increases in wildtype mice following exposure to pups but not to novel objects (Jin et al., 2005). Pups born to mice lacking the two major CREB isoforms, α and Δ (*Creb*-αΔ^−/−^) died within several days of birth, but the *Creb*-αΔ^−/−^ females were capable of rearing pups whose maternal care was initiated by wildtype females, demonstrating the complex role of CREB in maternal function.

The *Mecp2* gene that plays an important role in the repression of gene transcription was also linked to maternal behavior. The MECP2 protein binds to methylated DNA and controls transcriptional programs by modifying chromatin structure regulating plasticity in development and adulthood (Chahrour et al., 2008). Female heterozygous mutants of the *Mecp2* gene failed to show maternal retrieval when exposed to pups (Lau et al., 2020). Given that MECP2 is suggested to be involved in the behavioral response to chronic stress and in ketamine’s antidepressant effects (Uchida et al., 2011; Kawatake-Kuno et al., 2021; Kim et al., 2021), MeCP2 dysfunction may also be associated with PPD.

### Immediate Early Genes

Neuronal activation-dependent molecular signals must be relayed from active synapses to the nucleus to initiate activity-dependent gene transcription (Greer and Greenberg, 2008; Panayotis et al., 2015). Neuronal activity causes rapid expression of IEGs that are crucial for experience-driven changes in synapses and at the nucleus which are part of long-term intracellular changes ultimately leading to learning and memory. Some of these processes may very well be employed during social behaviors, such as maternal care. IEGs are the first genes to be activated following environmental stimulation. The transcription of IEGs is induced rapidly without a requirement for new protein synthesis (Yap and Greenberg, 2018). It however requires a neurotransmitter-induced influx of extracellular calcium into the neuron. The resulting increase in cytoplasmic calcium stimulates a cascade of signaling events, including the activation of the Ras-mitogen-associated protein kinase (MAPK), calcium/calmodulin-dependent protein kinases (CaMKs), and calcineurin-mediated signaling pathways. The first IEG shown to be involved in maternal care was *fosB* (Brown et al., 1996). FosB is an AP-1 transcription factor homologous to c-fos, and* FosB* knockout female mice showed reduced retrieving behaviors and a majority of the pups died before weaning (Brown et al., 1996). Similar to the c-Fos, FosB expression is also induced in the mPOA neurons during parenting (Brown et al., 1996; Kalinichev et al., 2000; Kuroda et al., 2007). Moreover, ERK phosphorylation and FosB induction in the mPOA were significantly attenuated by pharmacological blockade of ERK signal, suggesting that the ERK-FosB signaling has an important role in the initiation of parental behavior (Kuroda et al., 2007). Thus, maternal nurturing may require the fine tuning of the ERK-FosB signaling in the mPOA.

Transcriptomic profiling has demonstrated that similar to the learning processes, hundreds of genes are activated during pregnancy and the postpartum period (Kinsley et al., 2008; Ray et al., 2015; Gammie et al., 2016). Several hundred mouse genes were found to undergo transcriptional changes during pregnancy and the postpartum period compared to virgin females in several major brain areas responsible for maternal care, including the mPOA (Driessen et al., 2014), mPFC (Eisinger et al., 2014), lateral septum (Eisinger et al., 2013), and NAc (Zhao et al., 2014). Transcriptional changes in several hundred genes were also found in the hypothalamus, HPC, neocortex and cerebellum in pregnant and postpartum female mice compared to virgin females (Ray et al., 2015).

## Clinical Implications

PPD is a type of major depression that emerges in the perinatal period and can persist after the baby is born for several months. The depressive symptoms that occur in the peripartum period have detrimental effects for the mother and the development of the child. Studying the molecular and cellular mechanisms in animal models of maternal care and its dysfunction has important implications for the rational design of psychological and pharmacological treatments. As described in this review, there is accumulating evidence from work on experience- and activity-dependent events in maternal behavior in rodents that suggests that similar processes may be at work in humans in the peripartum.

The critical importance of the synapse-nucleus connectivity and the role of the microtubule cytoskeleton to mental disease in humans are seen in clinical studies and animal models (Marchisella et al., 2016). Changes in tubulin expression are found in the HPC and prefrontal cortex of psychiatric patients. Genetic linkage studies associate tubulin-binding proteins with an increased risk of developing schizophrenia and bipolar disorder. For many years, altered immunoreactivity of microtubule associated protein-2 (MAP2) has been a hallmark found in the brains of individuals with schizophrenia. Single nucleotide polymorphisms (SNPs) and increased mRNA have been identified for MAP6/STOP in the prefrontal cortex of patients with schizophrenia (Shimizu et al., 2006). Because MAP6/STOP gene knockout female mice demonstrate deficits both in maternal care and behaviors related to the “schizophrenia-like” phenotype, there is a possibility that this gene might also be involved in human maternal care.

Moreover, the analysis of the top 700 maternal genes against genes from autism, bipolar disorder and schizophrenia databases found overlapped genes for each of these three disorders (Eisinger et al., 2014; Gammie et al., 2016). Importantly, there is an increased incidence of bipolar disorder in mothers (Spinelli, 2009). Given that these disorders include social deficits, these genes warrant a follow up in both animal and human studies. However, while many molecules have been found to be changed in the peripartum, only a few have been studied in detail in maternal care. This demonstrates that the in-depth molecular and cellular studies of maternal care are in their infancy.

As discussed in the previous sections, GABARs are among the few genes that have been examined in significant detail and shown to have a critical role in maternal care and its dysfunction both in rodents and humans. Changes in GABARs are associated with neuroendocrine disruptions during PPD, and neuroactive steroids such as allopregnanolone can affect GABAergic signaling by modulating GABA_A_ receptors (Walton and Maguire, 2019). Lower levels of serum allopregnanolone can predict symptoms of PPD in women (Osborne et al., 2017) and the relationship between the GABA and allopregnanolone is important in the pathophysiology of PPD (Deligiannidis et al., 2019). The discovery of the GABAR deficiency and its connection to progesterone has allowed the development of a drug that is approved to use for PPD by the FDA (Mody, 2019). The first FDA-approved drug to treat PPD is allopregnanolone (Brexanolone), a naturally occurring neurosteroid which is made from the progesterone. Women with PPD on Brexanolone display a reduction of the depressive symptoms compared to the placebo group (Kanes et al., 2017; Meltzer-Brody and Kanes, 2020). In addition to pharmacological approaches, the knowledge gained from the molecular and cellular studies can be employed in development of sophisticated new designs of psychotherapy. More research is needed on specific trafficking, synaptic and transcriptional mechanisms to find better treatments of maternal dysfunction.

## Conclusion

We discussed in this review evidence that activity-dependent intracellular mechanisms known to control memory processes (Kandel et al., 2014; Poo et al., 2016) may also be critical for maternal care. The mechanisms underlying memory processes have also been suggested to regulate the behavioral adaptations found in autism spectrum disorders (ASD), drug addiction and social learning (Ebert and Greenberg, 2013; Nestler, 2013; Basu and Siegelbaum, 2015; Leblanc and Ramirez, 2020). Indeed, some of the genes regulating maternal care overlap with genes involved in ASD, reward and drug addiction (Gammie et al., 2016)—which is not surprising since offspring are highly rewarding to mothers, maternal care is a social behavior and changes in social behavior are a hallmark of ASD. While progress is being made to investigate molecular and cellular mechanisms, there has been no systematic approach to examine possible experience-dependent molecular events supporting maternal behavior. An additional level of complexity is that these activity-dependent mechanisms might be different depending on the brain regions involved. For example, genes enriched in core maternal regions regulating pup retrieval may not overlap with those enriched in cortico-limbic threat-related regions regulating affective postpartum behaviors. Future studies will be necessary to delineate how activity-dependent intracellular events regulate maternal behavior.

## Author Contributions

All authors contributed to writing of the manuscript. IF, FC, and GS reviewed the final form. All authors contributed to the article and approved the submitted version.

## Conflict of Interest

The authors declare that the research was conducted in the absence of any commercial or financial relationships that could be construed as a potential conflict of interest.

## Publisher’s Note

All claims expressed in this article are solely those of the authors and do not necessarily represent those of their affiliated organizations, or those of the publisher, the editors and the reviewers. Any product that may be evaluated in this article, or claim that may be made by its manufacturer, is not guaranteed or endorsed by the publisher.
